# Society Meetings

**Published:** 1892-08

**Authors:** 


The Lake Erie Medical Society held its regular quarterly meeting at the residence of Dr. J. G. Thompson, Angola, N. Y., on Friday, July 15, 1892. The following-named members were present:

Dr. T. H. Johnston, Brant; Dr. G. B. Stocker, Angola; Dr. F. E. Tuttle, Leon; Dr. George Lattin, Cattaraugus; Dr. N. G. Richmond, Fredonia; Dr. J. J. Sharp, Silver Creek; Dr. C. C. Johnson, Gowanda; Dr. M. B. Shaw, Eden; Dr. D. G. Alling, Dunkirk; Dr. R. M. Evarts, Irving; Dr. J. Cherry, Eden; Dr. W. A. Putnam, Westfield; Dr. J. G. Rugg, Gowanda; Dr. W. J. French, Hamlet; Dr. A. D. Lake, Gowanda; Dr. D. D. Loop, North East; Dr. E. E. Davis, Forestville; Dr. William Teft, South Dayton; Dr. W. M. Ward, North Collins; and Dr. J. G. Thompson, Angola.

Dr. B. H. Daggett and Dr. William Warren Potter, of Buffalo, were present by invitation.

The following list of papers was read and discussed, namely: Gout, by Dr. C. C. Johnson, of Gowanda; Infantile Indigestion, by Dr. W. M. Ward, of North Collins; Urethritis, its Treatment by Hot Water Irrigation, by Dr. B. H. Daggett, of Buffalo, by invitation; Puerperal Fever, by Dr. E. E. Davis, of Forestville.

The group of papers read was interesting, and the discussions were able and instructive. This active and progressive Society is to be commended upon the useful work that it performs. The Society was entertained, on this occasion, by Dr. and Mrs. Thompson, and it is almost unnecessary to add that the social and intellectual features of the meeting kept pace with each other in a neck-and-neck race.

The officers of the Society for the present year are: R. E. Moss, M. D., President, Gowanda; J. Cherry, M. D., Vice-President, Eden; William Teft, M. D., Secretary and Treasurer, South Dayton; Committee on Programme, George Lattin, M. D., Cattaraugus; C. C. Johnson, M. D., Gowanda.

